# Comparing the efficacy and side-effects of PDLASTA® (Pegfilgrastim) with PDGRASTIM® (Filgrastim) in breast cancer patients: a non-inferiority randomized clinical trial

**DOI:** 10.1186/s12885-021-08197-6

**Published:** 2021-04-23

**Authors:** Safa Najafi, Maryam Ansari, Vahid Kaveh, Shahpar Haghighat

**Affiliations:** 1 Breast Cancer Research Center, Motamed Cancer Institute, ACECR, Tehran, Iran; 2 Department of Hematology and Medical Oncology, Firoozgar Hospital, Faculty of Medicine, Iran University of Medical Sciences, Tehran, Iran

**Keywords:** Pegfilgrastim, Filgrastim, Chemotherapy, Breast cancer, Iran

## Abstract

**Background:**

The objective of this study was to compare the efficacy and side effects of a single dose (Pegfilgrastim or PDL) or repeated six daily injections (Filgrastim or PDG) during chemotherapy courses in breast cancer patients in a non-inferiority clinical trial.

**Methods:**

In this randomized clinical trial, 80 patients were recruited and allocated randomly to two equal arms. In one group, a single subcutaneous dose of PDL was injected the day after receiving the chemotherapy regimen in each cycle. The second arm received a subcutaneous injection of PDG for six consecutive days in each cycle of treatment. The side effects of GCF treatment and its effect on blood parameters were compared in each cycle and during eight cycles of chemotherapy.

**Results:**

Hematologic parameters showed no significant differences in any of the treatment courses between the two study groups. The comparison of WBC (*p* = 0.527), Hgb (*p* = 0.075), Platelet (*p* = 0.819), Neutrophil (*p* = 0.575), Lymphocyte (*p* = 705) and ANC (*p* = 0.675) changes during the eight courses of treatment also revealed no statistically significant difference between the two study groups. Side effects including headache, injection site reaction and muscle pain had a lower frequency in patients receiving PDL drugs.

**Conclusion:**

It seems that PDL is non-inferior in efficacy and also less toxic than PDG. Since PDL can be administered in a single dose and is also less costly, it can be regarded as a cost-effective drug for the treatment of chemotherapy-induced neutropenia.

**Trial registration:**

IRCT20190504043465N1, May 2019.

## Background

G-CSF is the main cytokine for the control of neutrophil production that is clinically used for the treatment of congenital and acquired neutropenia [1]. This cytokine increases the number of circulating neutrophils in vitro and improves their performance [2]. More than 90% of patients respond to G-CSF by an increase of more than 1 × 10^9^/L in ANC (Absolute Neutrophil Count) [3, 4]. These patients benefit greatly from G-CSF [4, 5]; for example, by showing a significant improvement in their quality of life, including health, performance in society and socioeconomic status, a reduction in the frequency and severity of infections, fever, use of antibiotics, hospitalization and oral ulcers and an increased survival rate [5–12]. Furthermore, treating children with severe congenital neutropenia reduces the risk of sepsis severely [13]. This cytokine improves the quality of life of patients significantly. Treatment with rhG-CSF improves all previous chronic infections, decreases the frequency of new episodes of infection, and helps discontinue the administration of prophylactic antibiotics [6]. Various studies have been conducted on the side effects of G-CSF. Increased spleen size has been reported as a side effect in most patients. Also, the effect of G-CSF on bone marrow stimulation and development is manifested as early bone pain [8]. According to the Severe Chronic Neutropenia International Registry (SCNIR), the side effects of these patients include bone pain, splenomegaly, thrombocytopenia, osteoporosis, and leukemia/MDS [4, 14] as well as fever, myalgia, and erythema [15]. G-CSF has several effects on the granulocytic cell line; not only does it stimulate the growth and differentiation of myeloid precursors, it also enhances the activity of adult neutrophils [16]. According to numerous studies, the side effects of this drug include splenomegaly, thrombocytopenia, osteopenia and osteoporosis, bone pain, vasculitis, skin rash, eosinophilia, monocytosis and malignant changes to AML/MDS [2–4, 7, 10, 17–19]. Other studies with disparate findings have reported symptoms such as hyperplasia, glomerulonephritis, myalgia, erythema, dyspnea, hypotension, sweating, and hot flashes [2, 15, 20]. The most important of these complications is the progress of MDS to AML, although it is still unclear whether G-CSF is the cause of this progression or if the increased survival of congenital patients by G-CSF creates an opportunity for this transformation to take place because of the inherent tendency of MDS to progress towards the congenital neutropenic disease called AML [21, 22]. Various cytogenetic abnormalities have been associated with these malignancies; for example, CSF3R mutation (the G-CSF receptor), ELA2 gene mutation, rascogenic activity, chromosome 7 monosomy, and chromosomal changes. These patients show resistance to G-CSF therapy and may develop severe infections, which are often life-threatening. These patients also often do not show good treatment outcomes even after hematopoietic stem-cell transplantation [3, 23, 24].

Most patients should take some kind of GCSF during dose-dense treatments. If receiving treatment with PGL, the patient should receive the drug every day after chemotherapy for at least six days, but if receiving PDL, only one shot of the drug is required in each course of chemotherapy. Therefore, the objective of this study is to compare the effectiveness and side effects between the two drugs in breast cancer patients in a non-inferiority clinical trial.

## Methods

**Fig. 1:**
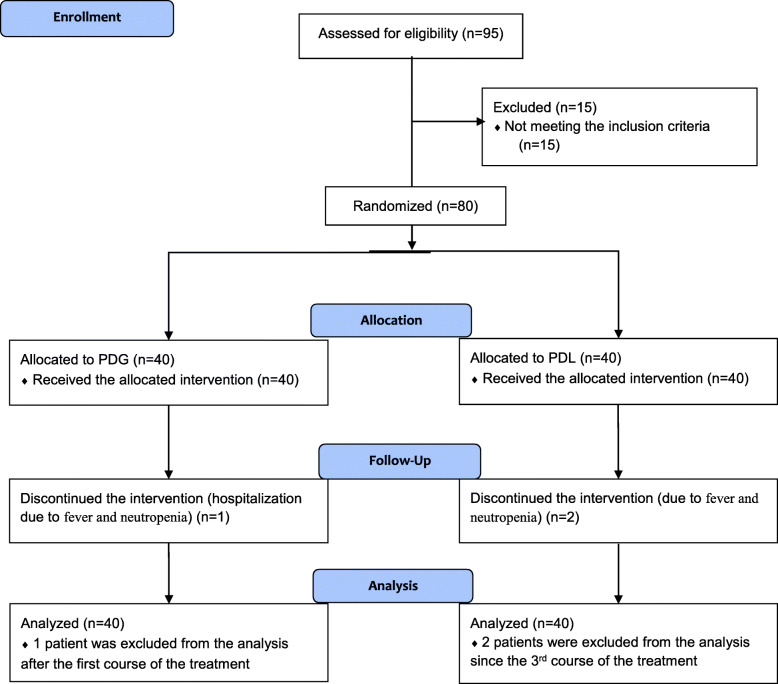
The CONSORT flow diagram

This interventional study compared the efficacy and safety of PDL produced by Pooyesh Darou Biopharmaceuticals Company with PDG in breast cancer patients as a non-inferiority, parallel-group, randomized clinical trial. Figure 1 presents the flow diagram of the study.

### Patients and criteria

This study was conducted on 80 patients diagnosed with breast cancer and referred to Motamed Cancer Institute for chemotherapy. They were treated with adjuvant and neoadjuvant therapy with a dose-dense AC*4-T*4 regimen consisting of four courses of Adriamycin plus Cyclophosphamide and four courses of Taxane-based drugs.

The inclusion criteria were:

- Age > 18 years
- Investigator diagnosis of breast cancer and being a candidate of adjuvant therapy
- Absolute neutrophil count ≥1.5 × 10^9^/l
- Platelet count ≥100 × 10^9^/l
- Serum creatinine < 1.5 × upper limits of normal
- Indication of receiving GCSF

The exclusion criteria consisted of:

- Bilirubin >upper limit of normal; or aspartate transaminase and/or alanine transaminase > 1.5 × upper limits of normal, concomitant with alkaline phosphatase > 2.5 × upper limit of normal
- Radiation therapy within 4 weeks of randomization into this study
- Prior bone marrow or stem cell transplantation
- Total lifetime exposure to doxorubicin > 240 mg/m^2^ or epirubicin > 600 mg/m^2^
- Ejection fraction < 40%
- Liver cirrhosis

In the case of serious complications due to the use of PDL, the patient was excluded from the study.

The eligible breast cancer patients who were under adjuvant and neoadjuvant chemotherapy with a dose-dense AC*4-T*4 regimen signed informed consent forms and entered the study. They were randomly assigned to the drug group (PDL) or to the control group (PDG) and their demographic and clinical characteristics were recorded. These drugs were administered to them free of charge under the supervision of a physician at the treatment center. Both groups received up to eight courses of chemotherapy, the first four of which consisted of Adriamycin and the second four of taxane-based drugs. Hematologic parameters and potential side effects of the drug were recorded based on the patients’ blood cell count and symptoms at baseline and on days 7 and 15 of each chemotherapy course.

The incidence of febrile neutropenia in any cycle was taken as a primary efficacy outcome. According to Holmes’ study [25], the rate of success of PDL and PDG in decreasing the incidence of neutropenia was 0.91 and 0.82, respectively. Considering the non-inferiority margin of 0.1, allocation ratio of 1/1, α error of 0.05, and 80% test power in this study, 40 patients were ultimately examined.

The patients were trained to inform the researchers of any complications they developed by calling the phone numbers given in the consent form. If any patients had fever and neutropenia in the first week or second week after the treatment, at least three additional doses of appropriate antibiotics were administered to them, and they were admitted to the hospital upon the physician’s advice. In patients with grade 3 or 4 of neutropenia, PDL would change to PDG until the end of the treatment and the patients were excluded from the PDL group. If the patient was hospitalized, Pooyesh Darou Biopharmaceuticals Company would incur all the treatment costs.

### Intervention

The patients in the PDL group received a single subcutaneous injection of 6 mg Pegfilgrastim on the second day of each chemotherapy cycle. In the PDG group, in each chemotherapy cycle, 300 micrograms Filgrastim per day was injected subcutaneously for six consecutive days.

### Outcome

The outcomes of interest were hematologic parameters, including WBC,^1^ Hgb,^2^ Platelet, Neutrophil, Lymphocyte, and ANC,^3^ and their values were compared between the two groups in each of the eight courses of chemotherapy. ANC was calculated by multiplying the percentage of neutrophils by the total number of WBCs (in thousands). The short and long-term side effects of the drugs were recorded in both groups during the study.

### Randomization and blinding

Randomization was performed using quadruple blocks. The randomization blocks were pockets given by the corresponding researcher. The blinding procedure was supervised by a staff member of the clinic who was not involved in the patients’ enrolment. The oncologist assigned the participants to the interventions. Due to the different administration protocols of the two drugs and the need for the supervision of an oncologist, blinding the patients and therapists was not possible. The statistical analyst was also not informed about the assignment of the patients to the groups.

### Statistical analysis

An interim analysis was achieved after reaching one-third of the sample size. Since no side effects were noticed in the two groups, recruitment continued until the end of the study. Descriptive statistics were used to present the frequency of the demographic and clinical characteristics in the two groups. The randomized allocation of the participants into the two groups was assessed using the Chi-square test and the student’s T-test. The Kolmogorov-Smirnov test was applied to evaluate the normality of the outcome variables’ distribution. Most variables did not have a normal distribution and nonparametric tests were therefore used in the next steps of the analysis.

The mean and median of the distribution of the hematologic variables (WBC, HgB, platelet, neutrophil**,** lymphocyte, and ANC**)** and the frequency of complications were compared between the PDG and PDL groups.

Changes in hematologic variables from baseline until days 7 and 15 in each course of chemotherapy were evaluated in both groups using Friedman’s test. The variation in repeated measurements of the outcome was compared between the two groups using Generalized Estimating Equation (GEE) analysis. The changes in the hematologic parameters during the eight courses of chemotherapy were compared between the two groups using the GEE analysis as well. The statistical analyses of the data were performed in SPSS software version 22.

### Ethical considerations

The patients entered the study by signing a written informed consent for drug intake. All information such as emphasizing the process of implementation, the right to withdraw from the study during the treatment, covering expenses, possible side effects and an emergency phone number for consultation and reporting side effects were included in the informed consent form. This research was approved by the Ethics Committee of the Breast Cancer Research Center with the code: IR.ACECR.IBCRC.REC.1395.19. The research project was also registered at the Iranian Registry of Clinical Trials (IRCT) in https://www.irct.ir/ with the registration code: IRCT20190504043465N1.

## Results

Eighty breast cancer patients were equally allocated to the PDL and PDG groups. Table 1 compares the demographic and clinical characteristics of the patients treated by PDG or PDL. The mean age of the PDG and PDL groups was 47.8 ± 9.04 and 43.7 ± 9.23, respectively. There was no significant difference between the two groups in terms of age, BMI,^4^ tumor size, excised LN,^5^ involved LN, Ki-67 index, education, marital status, employment status, ER,^6^ PR,^7^ and HER2.^8^

**Table 1:** The demographic and clinical characteristics of the patients in the two study groups

Variable	PDG	PDL	P-value
	Mean ± SD	Mean ± SD	
**Age, year**	47.8 ± 9.04	43.7 ± 9.23	0.05
**BMI, kg/m**^**2**^	27.8 ± 4.88	26.6 ± 4.06	0.261
**Tumor size, cm**	3.0 ± 1.1	3.1 ± 0.9	0.744
**Excised LN, n**	7.9 ± 4.6	8.7 ± 6.1	0.566
**Involved LN, n**	2.1 ± 2.7	2.1 ± 2.6	0.958
**Ki-67, %**	36.0 ± 23.2	34.6 ± 26.5	0.819
	**No (%)**	**No (%)**	
**Age**			0.116
< 50	24 (60)	30 (75)	
≥ 50	16 (40)	10 (25)	
**Education**			0.362
Illiterate/Primary School	16 (42.1)	19 (48.7)	
High School Diploma/University Education	22 (57.9)	20 (51.3)	
**Marital status**			0.387
Married	33 (82.5)	33 (87.5)	
Single/Divorced/Widowed	7 (17.5)	5 (12.5)	
**Employment status**			0.293
Housewife	30 (75.5)	33 (82.5)	
Employed	10 (25)	7 (17.5)	
**ER**			0.150
Negative	11 (28.2)	6 (15.8)	
Positive	28 (71.8)	32 (84.2)	
**PR**			0.061
Negative	19 (48.7)	11 (28.9)	
Positive	20 (51.3)	27 (71.1)	
**HER2**			0.587
Negative	31 (79.5)	30 (78.9)	
Positive	8 (20.5)	8 (21.1)	

Outcome measurements were achieved at baseline and on days 7 and 15 in each course of chemotherapy. Table 2 presents the distribution of the hematologic variables in the eight courses of chemotherapy in both groups.

**Table 2:** The changes in hematologic variables during the eight courses of chemotherapy in the two groups

Time	Variable	PDG Mean ± SD	PDL Mean ± SD
**Baseline**	WBC	9068.42 ± 1433.59	8361.58 ± 1992.03
	Hgb	12.75 ± 1.04	12.98 ± 1.2
	Platelet	298,972.22 ± 130,889.81	281,910.53 ± 104,249.08
	Neutrophil	74.5 ± 6.62	75.37 ± 6.09
	Lymphocyte	24.97 ± 6.38	22.92 ± 6.92
	ANC	6659 ± 1263	6388 ± 1682
**1**_**st**_ **course (day** 7**)**	WBC	3623.68 ± 1272.46	5997 ± 12,404
	Hgb	12.56 ± 1.01	12.8 ± 1.01
	Platelet	141,722.22 ± 77,288.18	136,584 ± 32,865
	Neutrophil	46.5 ± 6.19	47.6 ± 6.3
	Lymphocyte	52.97 ± 6.57	51.3 ± 6.6
	ANC	1662 ± 737	2896 ± 6062
**1**_**st**_ **course (day** 15**)**	WBC	5618.42 ± 1216.74	5827.63 ± 1127.22
	Hgb	12.52 ± .95	12.76 ± 1.107
	Platelet	182,694.44 ± 35,740.19	185,578.95 ± 32,961.59
	Neutrophil	85.18 ± 5.4	84.66 ± 5.54
	Lymphocyte	14.45 ± 4.75	15 ± 5.57
	ANC	4655 ± 1292	4891 ± 1054
**2**_**nd**_ **course (day** 7**)**	WBC	2786.84 ± 850.17	3553.16 ± 3202.25
	Hgb	12.36 ± 1.03	12.44 ± 1.06
	Platelet	109,055.55 ± 19,608.95	117,118.42 ± 20,749.29
	Neutrophil	47.71 ± 6.21	50.52 ± 8.98
	Lymphocyte	51.23 ± 6.09	48.21 ± 10.82
	ANC	1324 ± 476	1819 ± 1891
**2**_**nd**_ **course (day** 15**)**	WBC	5405.52 ± 5803.10	4693.42 ± 831.65
	Hgb	12.25 ± .96	12.4 ± 1.18
	Platelet	152,444.44 ± 12,622.98	160,131.58 ± 20,145.64
	Neutrophil	86.73 ± 5.75	86.34 ± 4.39
	Lymphocyte	14.02 ± 8.14	12.89 ± 3.67
	ANC	4550 ± 4351	4009 ± 808
**3**_**rd**_ **course (day** 7**)**	WBC	2610.52 ± 1345.12	2660.52 ± 1277.09
	Hgb	12.14 ± .93	15.23 ± 17.99
	Platelet	97,802.78 ± 14,290.07	103,476.31 ± 132,901.69
	Neutrophil	47.4 ± 6.9	51.18 ± 4.57
	Lymphocyte	51.97 ± 6.52	47.55 ± 5.25
	ANC	1215 ± 582	1359 ± 636
**3**_**rd**_ **course (day** 15**)**	WBC	3961.31 ± 668.86	3993.68 ± 717.65
	Hgb	12.06 ± .92	15.14 ± 17.99
	Platelet	142,861.11 ± 14,204.93	145,589.47 ± 27,305.87
	Neutrophil	87.1 ± 5.05	87.87 ± 4.47
	Lymphocyte	12.81 ± 5.11	12 ± 4.6
	ANC	1864 ± 431	2010 ± 450
**4**_**th**_ **course (day** 7**)**	WBC	2284.47 ± 1369.58	1976.58 ± 380.76
	Hgb	11.92 ± .92	12.07 ± .84
	Platelet	90,383.33 ± 28,554.37	92,842.1 ± 11,083.29
	Neutrophil	49.1 ± 10.91	50.26 ± 4.92
	Lymphocyte	50.29 ± 10.68	47.68 ± 6.63
	ANC	1148 ± 881	3711 ± 968
**4th course (day** 15**)**	WBC	4213.15 ± 4968.81	3415.79 ± 789.98
	Hgb	11.61 ± 1.95	11.98 ± .78
	Platelet	131,355.55 ± 27,377.83	134,000 ± 27,496.44
	Neutrophil	86.52 ± 7.29	86.79 ± 4.89
	Lymphocyte	13.34 ± 7.21	13.08 ± 4.74
	ANC	3563 ± 3892	996 ± 222
**5**_**th**_ **course (7th day)**	WBC	2593.42 ± 538.79	2455.52 ± 688.89
	Hgb	11.78 ± .93	11.93 ± .79
	Platelet	104,027.78 ± 12,112.53	100,473.68 ± 29,235.82
	Neutrophil	50.53 ± 6.55	52.63 ± 5.54
	Lymphocyte	47.79 ± 9.516	46.55 ± 6.41
	ANC	1312 ± 360	1299 ± 406
**5**_**th**_ **course (15th day)**	WBC	5339.47 ± 1450.54	5121.08 ± 1699.03
	Hgb	11.68 ± .96	11.89 ± .76
	Platelet	153,888.89 ± 21,372.58	158,657.89 ± 42,731.9
	Neutrophil	88.24 ± 6.32	88.58 ± 4.18
	Lymphocyte	11.63 ± 6.41	10.87 ± 3.4
	ANC	4681 ± 1328	4554 ± 1597
**6**_**th**_ **course (7th day)**	WBC	3515.79 ± 701.65	3531.58 ± 889.23
	Hgb	11.6 ± 1.03	11.85 ± .71
	Platelet	118,111.11 ± 22,193.66	116,078.95 ± 17,653.4
	Neutrophil	51.66 ± 6.84	52.6 ± 6.82
	Lymphocyte	47.55 ± 7.03	46.95 ± 7.17
	ANC	1809 ± 460	1879 ± 755
**6**_**th**_ **course (15th day)**	WBC	7200 ± 1567.23	7056.58 ± 1948.95
	Hgb	11.58 ± .9	11.76 ± .72
	Platelet	173,972.22 ± 19,983.54	171,236.84 ± 37,220.62
	Neutrophil	88.73 ± 5.23	88.37 ± 3.97
	Lymphocyte	11.13 ± 5.2	11.63 ± 3.97
	ANC	6371 ± 1558	6244 ± 1792
**7**_**th**_ **course (7th day)**	WBC	5722.37 ± 7130.64	4190.79 ± 757.38
	Hgb	11.52 ± .88	11.69 ± .73
	Platelet	124,611.11 ± 15,331.16	122,815.79 ± 16,204.53
	Neutrophil	52.87 ± 5.5	68.34 ± 98.78
	Lymphocyte	46.34 ± 5.82	46.18 ± 8.47
	ANC	3086 ± 4284	2923 ± 4491
**7**_**th**_ **course (15th day)**	WBC	11,797.37 ± 14,999.43	8007.9 ± 2431.03
	Hgb	11.48 ± .95	11.63 ± .76
	Platelet	183,500 ± 21,285.14	181,289.47 ± 31,302.28
	Neutrophil	89.44 ± 4.85	90.63 ± 3.83
	Lymphocyte	9.05 ± 3.38	10.64 ± 10.17
	ANC	10,515 ± 13,354	7273 ± 2273
**8**_**th**_ **course (7th day)**	WBC	5396.05 ± 1612.13	5010.53 ± 945.41
	Hgb	11.15 ± 1.91	11.51 ± .81
	Platelet	128,583.33 ± 20,230.63	127,594.74 ± 17,224.53
	Neutrophil	53.55 ± 6.03	52.21 ± 7.29
	Lymphocyte	45.39 ± 8.08	46.63 ± 7.65
	ANC	2924 ± 1057	2624 ± 648
**8**_**th**_ **course (15th day)**	WBC	13,300 ± 6704.21	13,448.68 ± 17,942.84
	Hgb	11.52 ± 1.24	11.41 ± .87
	Platelet	193,444.44 ± 26,144.04	194,526.31 ± 41,315.36
	Neutrophil	88.94 ± 7.64	90.05 ± 4.77
	Lymphocyte	10.68 ± 7.39	9.71 ± 4.79
	ANC	11,861 ± 6426	12,133 ± 16,165

**Fig. 2:**
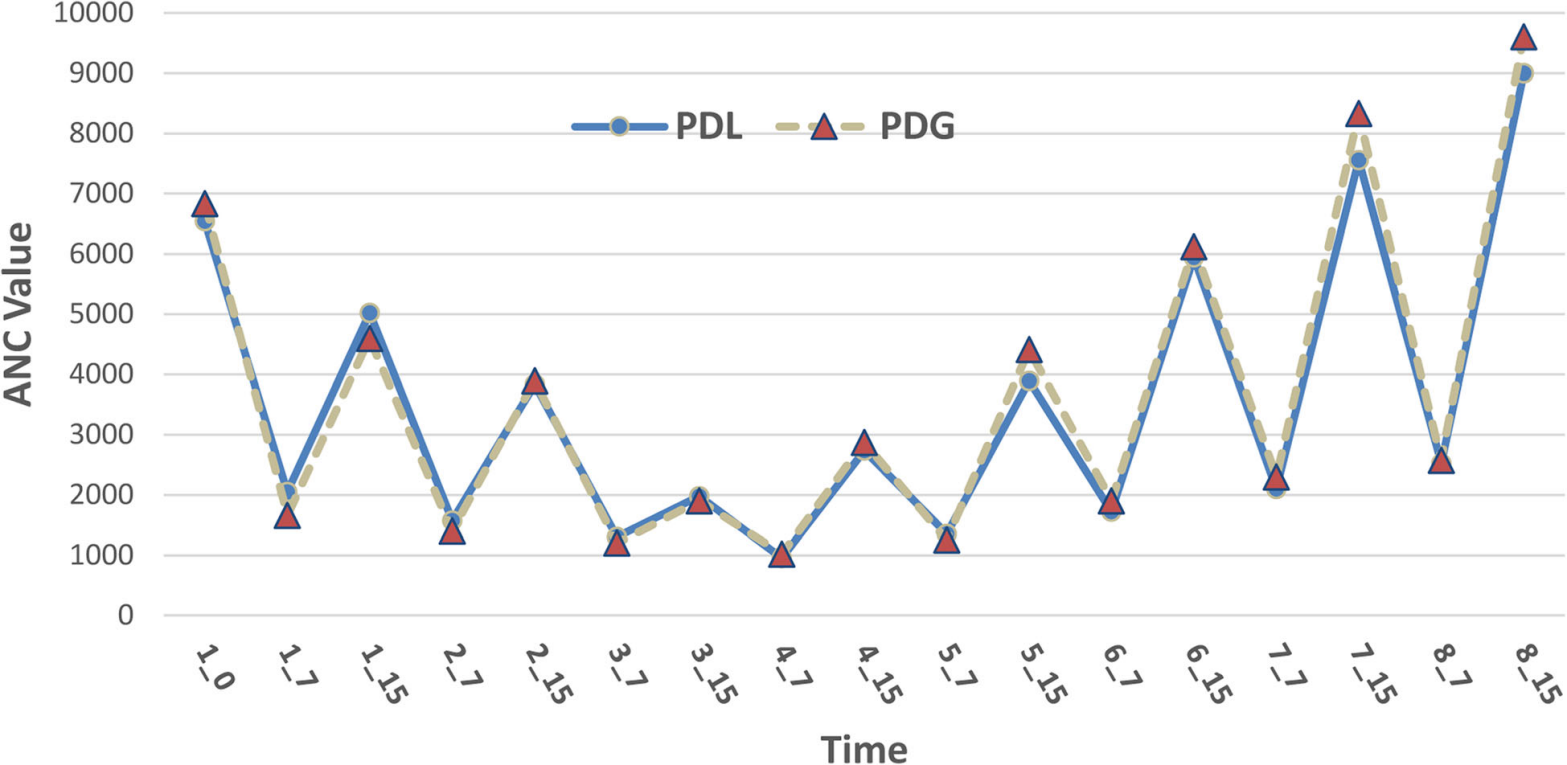
Changes in ANC values during the eight courses of chemotherapy in the two groups

Figure 2 shows the changes in ANC values during the eight courses of chemotherapy in the PDG and PDL groups. Similar trends of ANC values are noticeable between the two groups.

The within- and between-groups variations in blood count were analyzed at baseline and on days 7 and 15 in each course of chemotherapy (Table 3). The results suggest that, in both the PDL and PGL groups, all the hematologic components (WBC, Hgb, Plt, Neut, Lymph and ANC) changed significantly during each course of chemotherapy. A reduction in the hematologic component and its increase after GCF injection was the prominent pattern of data variations in each course. Nonetheless, in the fifth course of PDL injection, Hgb showed no significant changes (*P* = 0.095).

**Table 3:** Comparing the within- and between-group blood counts measured at baseline and on days 7 and 15 in each course of chemotherapy

Courses		PDL Group (Median)			p-value*	PGL Group (Median)			p-value*	p-value**
		D0	D7	D15		D0	D7	D15		
**1**	**WBC**	8900	4100	5850	< 0.001	9550	3850	5750	< 0.001	0.295
	**Hb**	13.20	13.00	13.00	< 0.001	12.80	12.35	12.25	< 0.001	0.159
	**Plt**	280,000	124,000	173,000	< 0.001	248,500	124,000	168,000	< 0.001	0.416
	**Neut**	75	50	85	< 0.001	70	50	85	< 0.001	0.312
	**Lymph**	25	50	15	< 0.001	27	50	15	< 0.001	0.130
	**ANC**	6545	2050	5025	< 0.001	6830	1660	4600	< 0.001	0.327
**2**	**WBC**	5850	3100	4400	< 0.001	5750	3000	4500	< 0.001	0.866
	**Hb**	13.00	13.00	12.90	< 0.001	12.25	12.20	12.20	< 0.001	0.307
	**Plt**	173,000	111,000	156,000	< 0.001	168,000	107,000	156,000	< 0.001	0.468
	**Neut**	85	50	85	< 0.001	85	50	86	< 0.001	0.504
	**Lymph**	15	50	13	< 0.001	15	50	14	< 0.001	0.243
	**ANC**	5025	1575	3838	< 0.001	4600	1400	3895	< 0.001	0.950
**3**	**WBC**	4400	2450	3950	< 0.001	4500	2500	3950	< 0.001	0.820
	**Hb**	12.90	12.90	12.30	< 0.001	12.20	12.00	12.00	< 0.001	0.107
	**Plt**	156,000	101,000	139,000	< 0.001	156,000	100,000	146,000	< 0.001	0.302
	**Neut**	85	50	90	< 0.001	86	50	90	< 0.001	**0.023**
	**Lymph**	15	50	10	< 0.001	14	50	10	< 0.001	**0.006**
	**ANC**	3838	1305	1975	< 0.001	3895	1200	1900	< 0.001	0.229
**4**	**WBC**	3950	1900	3100	< 0.001	3950	2100	3400	< 0.001	0.443
	**Hb**	12.30	12.05	12.05	< 0.001	12.00	12.00	12.00	< 0.001	0.473
	**Plt**	139,000	92,500	128,500	< 0.001	146,000	98,000	132,000	< 0.001	0.969
	**Neut**	90	50	90	< 0.001	90	50	90	< 0.001	0.317
	**Lymph**	10	50	11	< 0.001	10	50	10	< 0.001	0.143
	**ANC**	1975	955	2738	< 0.001	1900	1020	2872	< 0.001	0.548
**5**	**WBC**	3100	2500	4500	< 0.001	3400	2500	4900	< 0.001	0.341
	**Hb**	12.05	12.00	12.00	**0.095**	12.00	12.00	11.50	< 0.001	0.237
	**Plt**	128,500	100,000	151,000	< 0.001	132,000	102,000	159,000	< 0.001	0.974
	**Neut**	90	50	90	< 0.001	90	50	90	< 0.001	0.413
	**Lymph**	11	50	10	< 0.001	10	50	10	< 0.001	0.590
	**ANC**	2738	1350	3895	< 0.001	2872	1250	4410	< 0.001	0.289
**6**	**WBC**	4500	3200	6750	< 0.001	4900	3450	6800	< 0.001	0.581
	**Hb**	12.00	12.00	12.00	0.001	11.50	11.40	11.30	< 0.001	0.206
	**Plt**	151,000	111,500	159,500	< 0.001	159,000	110,000	174,000	< 0.001	0.919
	**Neut**	90	50	90	< 0.001	90	50	90	< 0.001	0.663
	**Lymph**	10	50	10	< 0.001	10	50	10	< 0.001	0.743
	**ANC**	3895	1725	5948	< 0.001	4410	1900	6120	< 0.001	0.543
**7**	**WBC**	6750	4200	8400	< 0.001	6800	4600	9100	< 0.001	0.050
	**Hb**	12.00	11.90	11.50	< 0.001	11.30	11.30	11.30	0.002	0.342
	**Plt**	159,500	121,000	170,500	< 0.001	174,000	124,000	186,000	< 0.001	0.559
	**Neut**	90	50	90	< 0.001	90	50	90	< 0.001	0.306
	**Lymph**	10	50	10	< 0.001	10	50	10	< 0.001	0.898
	**ANC**	5948	2100	7560	< 0.001	6120	2300	8330	< 0.001	0.146
**8**	**WBC**	8400	4950	10,000	< 0.001	9100	5100	10,550	< 0.001	0.395
	**Hb**	11.50	11.35	11.35	< 0.001	11.30	11.25	11.25	< 0.001	0.528
	**Plt**	170,500	124,000	186,500	< 0.001	186,000	124,000	194,500	< 0.001	0.637
	**Neut**	90	50	90	< 0.001	90	50	90	< 0.001	0.781
	**Lymph**	10	50	10	< 0.001	10	50	10	< 0.001	0.838
	**ANC**	7560	2525	9000	< 0.001	8330	2575	9601	< 0.001	0.434

P-value*: Repeated measurements within the groups (Friedman’s test)

P-value**: Repeated measurements between the groups (GEE analysis)

Applying GEE analysis showed no significant differences between the trend of hematologic values during most courses of chemotherapy; however, there were two exceptions in the third course of chemotherapy, during which the neutrophil count (*p* = 0.023) and lymphocyte count (*p* = 0.006) had lower fluctuations in the PDL group.

The changes in hematologic parameters during the eight courses of chemotherapy were compared between the two groups using GEE analysis. The comparison of the WBC (*p* = 0.527), Hgb (*p* = 0.075), platelet (*p* = 0.819), neutrophil (*p* = 0.575), lymphocyte (*p* = 705) and ANC (*p* = 0.675) changes during the eight courses of treatment identified no statistically significant differences between the two study groups.

Since the probability of neutropenia frequency fluctuates during treatment, the changes in blood parameters were compared between the first four courses of chemotherapy and the second four courses. In the PDG group, the mean values of WBC, Plt, and ANC in the first half of treatment were 6281, 153,171, and 3358, and in the second half, they were 6280, 151,064 and 5220, respectively. The *P*-value for the mean difference in WBC, Plt and ANC between the two treatment halves was < 0.001, 0.543 and < 0.001. In the PDL group, the mean values of WBC, Plt, and ANC in the first half of the treatment were 4446, 154,095 and 3249, and in the second half, they were 6820, 152,298 and 5575, respectively. The P-value for the mean difference in WBC, Plt and ANC between the two treatment halves was < 0.001, 0.651 and < 0.001.

The results showed that the changes in WBC (*p* = 0.439), Hgb (*p* = 0.052), platelet (*p* = 0.7), neutrophil (*p* = 0.324), lymphocyte (*p* = 0.463) and ANC (*p* = 0.571) during the two halves of treatment did not differ significantly between the two study groups.

Table 4 presents a comparison of the side effects between the two groups. There was no side effect in 50% of the patients in the PDL group compared to 12.5% in the PDG group. The most common side effects in the PDG group were musculoskeletal pain, with a relative frequency of 47.5%, compared to 15% in the PDL group. Headache (30%), injection site reaction (25%), leukocytosis (20%), and bone pain (17.5%) were other common side effects in the PDG group.

**Table 4:** A comparison of very common side effects between the two groups

Side effect	PDG N (%)	PDL N (%)	Total N (%)
**No**	5 (12.5)	20 (50%)	25 (31.25)
**Headache**	24 (30)	10 (25)	34 (42.5)
**Bone pain**	7 (17.5)	3 (7.5)	10 (12.5)
**Nausea**	3 (7.5)	4 (10)	7 (8.75)
**Musculoskeletal pain**	19 (47.5)	6 (15)	25 (31.25)
**Fever**	–	1 (2.5)	1 (1.25)
**Injection site reaction**	10 (25)	1 (2.5)	11 (13.75)
**Leukocytosis**	8 (20)	4 (10)	12 (15)
**Non-cardiac chest pain**	1 (2.5)	–	1 (1.25)
**Anaphylaxis**	1 (2.5)	–	1 (1.25)

Three patients were excluded from the study. In the PDL group, two patients were excluded from the study because of fever and neutropenia. The first patient received PDL in two courses of chemotherapy. In the third course, ANC decreased to 490 on the 7th day of the injection. In the second case in this group, an ANC of 750 was recorded at the end of the third course of chemotherapy. Antibiotics were administered to both patients out-patiently, and PDL was not continued for them. As for the PDG group, one patient from this group was hospitalized because of fever and neutropenia after one course of injection. Her ANC in the first course of chemotherapy decreased from 5440 to 70 during 15 days of injection. She had oral mucositis and a high grade of fever. She recovered after three days of antibiotic therapy in the hospital.

## Discussion

All the chemotherapy regimens used in this study consisted of chemotherapy drugs that cause more than 10% neutropenia without GCF. The chemotherapeutic agents used in the study had eight courses. The first four courses were completely similar and composed of Doxorubicin and Cyclophosphamide, which cause neutropenia and fever more frequently. The next four courses consisted of only Docetaxel, which is much less likely than the previous courses to cause neutropenia. The results showed that the changes in WBC (*p* = 0.439), Hgb (*p* = 0.052), platelet (*p* = 0.7), neutrophil (*p* = 0.324), lymphocyte (*p* = 0.463) and ANC (*p* = 0.571) during the two halves of treatment did not differ significantly between the two study groups. The changes in hematologic parameters during the eight courses of chemotherapy did not show any statistically significant differences between the two study groups.

Several studies have proven the efficacy of PDG as a drug. Due to the number of daily injections of the drug, PDG was released slowly. The PEGylated form of this drug has been effective and safe according to clinical trial studies. The PEGylation of drugs improves their clinical value; for instance, it increases their solubility [26], protects them against enzyme degradation [27], decreases their renal clearance [28], causes physical and thermal stability [29], and increases the antigenicity and toxicity half-life [30]. PDL is a G-CSF quadrilateral conjugate formulation whose efficacy and safety are comparable with PDG [31–33]. The half-life of PDL is 12 times longer than the half-life of non-conjunctive drugs. Polyethylene glycol binding to G-CSF reduces renal secretion and prevents its proteolysis, resulting in an increase in drug levels up to 14 days after single-dose administration. Following regular chemotherapy regimens, the number of leukocytes and the appearance of CD34 in the peripheral blood occur faster and sooner after PDL than G-CSF [34]. PDG has been used in chemotherapy-induced neutropenic patients and has recently been used to treat children’s neutropenia as well [32]. In a study by Holmes et al. in 2002, in which 154 female breast cancer patients were enrolled, 129 patients received PDL and 25 received G-CSF. Five patients had unbearable side effects that resulted in the discontinuation of the drug; one of these patients developed renal insufficiency with a dose of 100 micrograms per kilogram, and four others developed the following side effects with a dose of at least 30 micrograms: Fever, diarrhea, nausea and dehydration. Other side effects observed in all the patients were mild to moderate bone pain similar to PDL and G-CSF (35%), and 7% of the patients needed to use narcotics to relieve their pain [24]. There was no side effect in 50% of the patients in the PDL group compared with 12.5% in the PDG group. Also, the most common side effects in the PDG group were musculoskeletal pain, with a 47.5% frequency compared to 15% in the PDL group, followed by injection site reaction, with a 25% frequency in the PDG group. Headache (30% vs. 25%), injection site reaction (25% vs. 2.5%), leukocytosis (20% vs. 10%), and bone pain (17.5% vs. 7.5%) were other common side effects in the PDG group. Since toxicity grades I and II do not change the treatment protocol and stratified analysis based on different grades of adverse effects would greatly increase the sample size, making a trial unfeasible, we considered grade-III and IV toxicities as positive side effects.

In a study conducted on 310 adjuvant chemotherapy patients taking 75 mg Docetaxel daily and 60 mg Doxorubicin per square meter of body surface area on the first day of each cycle for a maximum of four cycles, the patients who received 100 micrograms per kilogram of weight PDL were compared with the patients who received 5 micrograms per kilogram of weight PDG on the second day of the cycle were compared with each other. The results were almost similar in the two groups, and the ANC values ​​were not significantly different between the two groups, and neutropenia with fever was less common in the patients who took PDL. PDL was tolerated and the side effect profile of the two groups was similar [25]. In another study randomly comparing multiple doses of PDL with filgrastim in breast cancer patients, a PDL dose of 100 micrograms per kilogram of weight had good efficacy and a favorable side effect profile [24]. In a double-blind, phase-III trial with a fixed 6-mg dose of PDL, febrile neutropenia was less common than G-CSF (13% vs. 20%) [3]. Any chemotherapeutic regimen can cause neutropenia, but when the absolute neutrophil count reaches below 1000, there is a very high risk of febrile neutropenia and sepsis. Therefore, most researchers believe that GCF should be used to prevent a reduction in absolute neutrophil count to below 1000.

In a study carried out on women receiving chemotherapy during pregnancy who were administered G-CSF and PDL, there were no significant changes in the gestational age at delivery, embryonic anomalies or the birth weight of the baby; in these patients, myelopoiesis stopped at the first stage of growth (the promyelocyte/myelocyte stage) [17]. In a study by Calderwood et al. in 2001, splenomegaly was reported in all the patients while mild hyperplastic hypertrophy was observed in a few, and no short-term drug toxicity was reported for them [2]. The results of this study using the diagram of trend of changes in ANC and platelet count and lymphocyte count as the main indicators of the effectiveness of drugs showed no significant differences between the two groups. In this study, despite the various side effects recorded for the drug, headache, bone pain and injection site reaction were the most important and common side effects, which showed the highest frequency in both groups. Therefore, future studies on the subject are recommended to investigate these particular side effects.

## Conclusion

PDL is completely non-inferior in efficacy and also less toxic than PDG. Its prescription as a single-dose drug that is also less expensive makes it a cost-effective treatment for chemotherapy-induced neutropenia.

## Data Availability

The datasets used and analysed during the current study are available from the corresponding author on reasonable request.

## Footnotes

1 White Blood Cells

2 Hemoglobin

3 Absolute Neutrophil Count

4 Body Mass Index

5 Lymph Nodes

6 Estrogen Receptor

7 Progesterone Receptor

8 Human Epidermal Growth Factor Receptor 2
